# Seasonal changes in the abundance *Fusarium proliferatium*, microbial endophytes and nutrient levels in the roots of hybrid bamboo *Bambusa pervariabilis* × *Dendrocalamopsis grandis*

**DOI:** 10.3389/fpls.2023.1185449

**Published:** 2023-07-19

**Authors:** Lin Li, Yaxuan Wang, Cailin Yu, Shuying Li, Tiantian Lin, Shan Han, Tianhui Zhu, Shujiang Li

**Affiliations:** ^1^College of Forestry, Sichuan Agricultural University, Chengdu, China; ^2^National Forestry and Grassland Administration Key Laboratory of Forest Resources Conservation and Ecological Safety on the Upper Reaches of the Yangtze River, Chengdu, China

**Keywords:** bamboo, basal rot, seasonal variation, root nutrient elements, root microorganism

## Abstract

Plant root pathogens invade the soil around plant roots, disturbing the systemic balance, reducing plant defenses, and causing severe disease. At present, there are few studies on the severity of plant diseases caused by pathogen invasion in different seasons and how pathogens affect root microecology. In this study, we compared the levels of nutrients in the root tissues of the two groups of plants. We used 16S and ITS amplicon sequencing with Illumina NovaSeq 6000 to compare seasonal changes in the composition and structure of microbial communities from healthy roots of bamboo *Bambusa pervariabilis* × *Dendrocalamopsis grandis* and roots infected by the soilborne pathogen Fusarium proliferatum. We have found that the invasion of the pathogen led to a substantial decrease in nutrient elements in bamboo roots, except for nitrogen. The pathogen presence correlated with seasonal changes in the bamboo root microbiome and decreased bacterial richness in diseased plants. The root microbial community structure of healthy plants was more stable than that of their diseased counterparts. Furthermore, we identified the lesion area and relative abundance of *F. proliferatum* were significant predictors of disease progression. The potassium tissue content and the disease lesion area were identified as factors linked with the observed changes in the bamboo root microbiome. This study provides a theoretical foundation for understanding the seasonal dynamics *F. proliferatum*, an economically important soilborne pathogen of hybrid bamboo grown in Sichuan Province, China.

## 1 Introduction

Plant soil-borne diseases are spread by pathogens infecting the roots and stems of plants through the soil and cause yield and quality losses that threaten the ecology and economy of agriculture and forestry (Gao et al., 2014). Many economically important soil-borne plant pathogens are fungi, such as *Fusarium* spp., *Thielaviopsis* sp., *Gaeumannomyces graminis*, *Verticilium* spp., *Phytophthora* spp., *Pythium* spp., and *Rhizoctonia solani* (Otten and Gilligan, 2006; Pane et al., 2013; Aleandri et al., 2015). These pathogens overwinter in the soil, and when the weather and soil characteristics are optimal, the pathogens will reproduce, spread, and infect plant roots, causing root rots, wilt complex disease, red crown rot, seedling blight, and southern blight (Ochi, 2017; Sánchez-Espinosa et al., 2020; Choi et al., 2021; Zhang X. et al., 2021; Zitnick-Anderson et al., 2020; Mageshwaran et al., 2022). The occurrence of soil-borne diseases is affected by climate changes such as temperature and humidity. The changes in temperature and humidity in different seasons will lead to changes in the abundance of pathogens in the soil, thus affecting the occurrence of plant diseases (Serrano et al., 2020). The increase in minimum and maximum temperatures may contribute to the increase in the severity of plant diseases (Mohammed et al., 2018; Prahl et al., 2023). An in-depth understanding of the relationship between the abundance of pathogens and the severity of plant diseases in different seasons is beneficial to the control of plant diseases.

In addition to environmental factors, the outcome of the soilborne pathogen infection depends on the host plant and its microbiome (Carrion et al., 2019). Underground plant parts are colonized by diverse communities of microorganisms that typically enter plants through the root system and subsequently infiltrate neighboring plant tissues (Wang Z. et al., 2021). Numerous microbial taxa associated with the rhizosphere (soil adhering to roots), the rhizoplane (root surface), and the endosphere (root interior tissues) have been demonstrated to play an important role in plant resistance to pathogens and serve as the first (rhizosphere) and second (rhizoplane and endosphere) lines of defense for plants (Alvin et al., 2014; Ge et al., 2022). Root microorganisms can improve plant growth by promoting nutrient absorption and synthesizing important phytohormones. They can also act as biological control agents, indirectly improving plant growth and minimizing pathogen intrusion by producing antibiotics, competing with plant pathogens for nutrition, and inducing host systemic resistance (Khare et al., 2018; Ali et al., 2021). Pathogens enter the plant’s internal tissues, including vascular tissues and intercellular spaces, to obtain more nutrients to avoid harsh and fluctuating environmental conditions (Fatima and Senthil-Kumar, 2015). The growth activity of the host will be disturbed by the pathogen’s invasion. When the pathogen invades the host plant from the root, it breaks the system balance and promotes disease development, and the community of plant root microorganisms will change significantly (Li et al., 2021; Mannaa and Seo, 2021). For example, the pathogen *Ilyonectria mors-panacis* in the root system of *Panax notoginseng* with root rot was more abundant than that in the healthy roots of *P. notoginseng* (Wang P. et al., 2021). The bacterial diversity in the roots of healthy tobacco plants was more abundant than that in the roots of tobacco plants suffering from wilt (Ahmed et al., 2022). The pathogen invasion of plant roots can affect the nutrient balance of plant roots and the stability of microbial community structure and diversity. Current studies have not fully explored the potential effects of plant pathogen invasion on root nutrition and root microbial communities under different seasonal variations.

*Fusarium* sp. is one of the most important macrofungal genera distributed in the world (He et al., 2021; Shan et al., 2021; Yang et al., 2022). *Fusarium* can infect many kinds of plants (food crops, medicinal plants, economic crops, and ornamental plants), which can cause disease in plants and seriously limit plant growth (Nayaka et al., 2011; Erazo et al., 2021). *Fusarium* mainly inhabits soil in the form of chlamydospores. Mycelium penetrates the root and extends into the root tissue to infect the vascular bundle system of the host plant. *Fusarium* continually produces toxic metabolites and leads to systematic yellowing, wilting, and death of plants (Lievens et al., 2008; Tan et al., 2022). *Fusarium* mycelia and spores overwinter in rhizosphere soil in the winter (December-February). *Fusarium* invades the young root epidermis or wound of the host in the following spring (March-May). It colonizes and propagates in the root system and blocks vascular tissue in the summer (June-August). And the growth of *Fusarium* gradually enters a decline period in the autumn (September-November) (Kazan and Gardiner, 2018; Malik et al., 2018; Gálvez and Palmero, 2022). Plant diseases caused by *Fusarium* can lead to plant death in severe cases, reduce plant yields, and hinder the development of agriculture and forestry.

*Bambusa pervariabilis × Dendrocalamopsis grandis* is the main cultivated bamboo species, which is generated by crossing *Bambusa pervaiabilis* Mc-Clure (as the female parent) and *Bambusa grandis* Keng f. (as the male parent) and is used when returning farmland to forests and building ecological barriers in the Yangtze River basin (Peng et al., 2020). Due to the well-developed root system and dense branches and leaves of hybrid bamboo, it can be used for afforestation along rivers. It has the advantages of windbreak and sand fixation, soil and water conservation, and improvement of the ecological environment. Bamboo can be used for papermaking, handicrafts, musical instruments, etc., with high economic and social value. Bamboo can also prevent soil erosion, help maintain the biodiversity of forest land, and provide ecological services (Fang et al., 2021). In recent years, *B. pervariabilis × D. grandis* has suffered from the effects of a variety of fungi, which led to the occurrence of a variety of diseases, such as shoot blight (Zhu et al., 2009), wilt (Ma et al., 2008), and stem rot (Xie et al., 2016), and hindered the growth of hybrid bamboo. In June 2020, the hybrid bamboo basal rot caused by *Fusarium proliferatum* was found in Renshou County, Sichuan Province, China. The damaged area was about 68 hectares, the incidence rate was 34.8%, and approximately 5% of the hybrid bamboo died (Li et al., 2022). The occurrence and spread of hybrid bamboo fungal diseases have led to the death of many hybrid bamboo forests, which seriously threatens ecological and economic development.

To better understand the effects of pathogens on plant roots, we advanced three hypotheses stating that 1) the relative abundance of *F. proliferatum* fluctuates seasonally leading to variation in the severity of basal rot of bamboo, and 2) the invasion of the pathogen affects the absorption of nutrient elements by plant roots and disturbs the plant root microbiome, and 3) the key influencing factors affect changes in root microbial communities. We tested these hypotheses by exploring the relationship between pathogen abundance and disease severity in different seasons and identify the dynamic changes and differences in nutrient elements between healthy bamboo roots and those with basal rot in different seasons. We also compared the composition and structural differences of microbial communities between healthy and diseased bamboo roots and identified key factors influencing the root microbiome. The results of this study provide a theoretical foundation for understanding the seasonal dynamics *F. proliferatum*, an economically important soilborne pathogen of hybrid bamboo grown in Sichuan Province, China

## 2 Materials and methods

### 2.1 Experiment site

The experiment site was located in Huaning Village (29°41′N, 104°11′E) in Renshou County, Sichuan Province, China. The area is characterized by the humid subtropical monsoon climate, with an average annual temperature of 17.4°C, an average annual rainfall of 1009.4 mm, an average annual sunshine of 1196.6 h, and a frost-free period of 312 days. It is suitable for growing various bamboos species, including *Dendrocalamus latiflorus*, *Bambusa emeiensis*, *Phyllostachys violascens*, and hybrid bamboos that cover an area of 194 hectares.

Three plots, D1, D2, and D3, were established at the three vertices of an equilateral triangle with a side length of 20 m, and each plot was 20 m × 20 m. Similar approach was used to establish the H1, H2, and H3 plots for sampling healthy hybrid bamboo plants at a site located 1 km away from the disease area (**Figure S1A**). Using the “S” sampling method (Li et al., 2020), two hybrid bamboos with typical symptoms of basal rot were randomly selected from each of the plots D1, D2, and D3 and named D1-1, D1-2, D2-1, D2-2, D3-1, and D3-2, and the D samples were from diseased plants. Two healthy hybrid bamboos were randomly selected from each of the three plots H1, H2, and H3, and named H1-1, H1-2, H2-1, H2-2, H3-1, and H3-2, and the H samples were from healthy plants. Twelve plants were selected from six plots.

### 2.2 Collection of bamboo roots

According to the growth characteristics of hybrid bamboo and climate change, samples were collected in spring (April), summer (July), autumn (October) in 2021, and winter (January) in 2022 (**Figures S1B–I**). The air temperature and humidity of the healthy plants and diseased plants were recorded by an air temperature and humidity monitor (HOBO, USA) (**Table S1**). After removing the dead branches and fallen leaves on the surface of the soil, roots with soil were collected at a depth of 0-20 cm in a circular range with a diameter of 0.5 m centered on the bamboo trunk. The soil on the surface of the roots was washed clean with sterile water, and the water on the surface was absorbed by sterile filter paper, which was put into 50-mL sterile centrifuge tubes and stored in liquid nitrogen. At the same time, the incidence of hybrid bamboo plants in six plots (D1, D2, D3, H1, H2, and H3) was observed. The lesion of basal rot was long, shuttle-shaped, to rectangular, and the area of the lesion was calculated by measuring the length and width of the lesion (Miller and Johnson, 2014). The following equation was used to calculate the percentage of plaque area:


$$\text{Percentage of lesion area }=\;\frac{\text{lesion length}\times \text{lesion width}}{\text{lesion length}\times \text{bamboo girth length}}\times 100\%$$

### 2.3 Determination of root nutrient elements

Root nitrogen content was determined by the Kjeldahl method (Kjeltec™ 8200, Foss, Hilleroed, Denmark) (Liu et al., 2014). Root phosphorus content was determined by the molybdenum-antimony anti-absorbance photometric method (U-2900UV/VIS, Hitachi, Tokyo, Japan) (Janket et al., 2021). Root potassium content was determined by flame atomic absorption spectrophotometry (M410, Sherwood Scientific, Cambridge, UK) (Weng et al., 2022). Iron, copper, calcium, zinc, manganese, and magnesium contents in roots were determined by flame atomic absorption spectrometry (iCE™ 3300 AAS, Thermo Scientific, Waltham, MA, USA) (Fauziah et al., 1990; Mandizvo and Odindo, 2019; He et al., 2022).

### 2.4 DNA extraction and sequencing of root samples

The DNA of the collected hybrid bamboo roots was extracted using the CTAB method (Lutz et al., 2011), and the purity and concentration of DNA were detected by agarose gel electrophoresis. Using the genomic DNA diluted with sterile water to 1 ng/µl as a template, Bacterial 16S V3-V4 variable sequences 341F (5′-CCTAYGGGRBGCASCAG-3′) (Muyzer et al., 1993) and 806R (5′-GGACTACNNGGGTATCTAA-3′) (Caporaso et al., 2011) and fungal ITS1-5F amplification region ITS5-1737F (5′-GGAAGTAAAAGTCGTAACAAGG-3′) and ITS2-2043R (5′-GCTGCGTTCTTCATCGATGC3′) (Liu et al., 2022) primers were used for PCR amplification. The total volume of the PCR reaction was 30 µL, Phusion Master Mix (2×) 15 µL, PrimerF (1 µM) 1 µL (1 µM), PrimerR (1 µM) 1 µL (1 µM), gDNA (1 ng/µL) 10 µL (5–10 ng), ddH_2_O complement the 30 µL system. The reaction procedure was as follows: pre-denaturing at 98°C for 1 min; 30 cycles including (98°C, 10 s; 50°C, 30 s; 72°C, 30 s); 72°C, 5 min. The obtained PCR products were detected by 2% agarose gel electrophoresis (voltage 120 v, 30 min). The qualified PCR products were mixed, and then the PCR products were detected by 2% agarose gel electrophoresis, and the target bands were recovered by the Universal DNA purification and recovery kit. The NEB Next^®^ Ultra DNA Library Prep Kit (Illumina, San Diego, CA, USA) was used for library construction, and the Agilent 5400 was used for detection and Q-PCR quantification. After the library was qualified, the Illumina NovaSeq 6000 (Illumina, San Diego, CA, USA) was used for on-machine sequencing. The high-throughput sequencing raw data of root bacteria and fungi were uploaded to the NCBI database, SRA: PRJNA936465 and PRJNA936468.

### 2.5 Sequence analysis

The data of each sample was split from the Raw PE, spliced, and filtered, and chimera sequences were removed to get Effective Tags. The Uparse algorithm (Edgar, 2013) was used to cluster all the Effective Tags of all samples, and by default, the sequence was clustered into OTUs (Operational Taxonomic Units) based on 97% identity. The species annotation analysis of the OTUs sequence was carried out by the Mothur method and the SSUrRNA database (Quast et al., 2013) of SILVA138 (Wang et al., 2007) (the threshold was set at 0.8–1). The taxonomic information was obtained, and the community composition of each sample was counted at each taxonomic level: kingdom, phylum, class, order, family, genus, and species. The phylogenetic relationships of all OTUs representative sequences were obtained by comparing multiple sequences with MUSCLE (Edgar, 2004) software.

### 2.6 Bioinformatics analysis

Using NovoGene’s free online platform (https://magic.novogene.com/), the abundance of *Fusarium* fungus OTUs in the roots of healthy and diseased plants sampled in different seasons was counted. One-way ANOVA and Duncan (α = 0.05) tests were performed to study the differences in lesion area at the stem base of diseased bamboo plants across different seasons and the differences in root nutrient elements, microbial α diversity index, and relative abundance of *Fusarium* spp. in roots between the healthy and diseased plants sampled across different seasons. Taking disease, season, and their interaction as independent factors, the differences in root nutrient elements and root microbial community α diversity between healthy plants and diseased plants in different seasons were studied by the generalized linear model. All analyses were conducted by SPSS 22 (IBM Corporation, NY, United States) and GraphPad Prism v8.0.2 (Halifu et al., 2019). A principal component analysis (PCA) based on a standardized method was used to analyze the nutrient elements of roots. The beta diversity of root microorganisms was analyzed by principal coordinate analysis (PCoA) based on binary_jaccard distance. The single factor similarity analysis (ANOSIM) method was used to analyze the influence and significance of different seasons and diseases on the beta diversity of the root microbial community. The linear discriminant analysis effect size (LEfSe) method was used to analyze the biomarkers between root microbiomes with an LDA Score > 4. DESeq2 was used to analyze the difference in genus abundance between diseased plants and healthy plants in the same season (Acharya et al., 2019). The two tools Variance inflation factor (VIF) and biological and environmental analysis (BioENV) in R (Version 2.15.3) were used to analyze the internal influencing factors and the relationship between influencing factors and species abundance, exclude the autocorrelated influencing factors, and retain the influencing factors that have the greatest impact on flora. Canonical correlation analysis (CCA) and Spearman correlation analysis were used to analyze the relationship between root nutrient elements, disease incidence index, air temperature, air relative humidity, and root microbial groups. PICRUSt2 and FUNGuild were used to predict the function of bacterial and fungal OTUs, respectively. All microbiome analyses were conducted through NovoGene’s free online platform. An overview of the sequencing data was included in the **Supplementary Material** (**Figure S2**).

The sequences of potential *Fusarium* fungi OTUs were searched by nucleotide BLAST in the National Biotechnology Information Center (NCBI) database (GenBank) for sequence comparison, and the top five representative sequences with the highest similarity were downloaded. Alignment was manually edited as needed, multiple sequences were compared using ClustalW (Larkin et al., 2007), and downloaded sequences were aligned and cut using MEGA 11. The Evolview web server was used to view and edit the constructed phylogenetic tree (Subramanian et al., 2019).

## 3 Results

### 3.1 Changes in lesion area at the base of the bamboo stem and root nutrient elements

**Figure 1:**
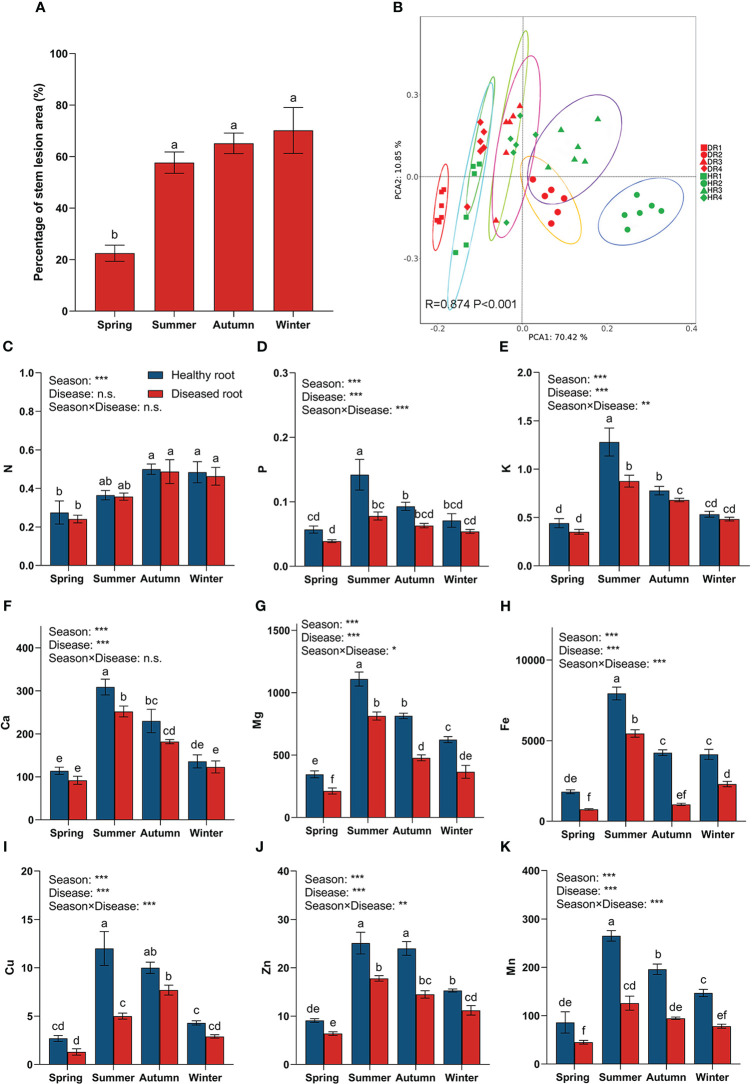
Percentage diagram of lesion area at the stem base of diseased plants in different seasons and the nutrient content of plant roots in different seasons. **(A)** Percentage diagram of lesion area of hybrid bamboo. **(B)** Principal component analysis (PCA) diagram of root nutrient elements, with an ellipse representing a 95% confidence interval. One-way similarity analysis (ANOSIM) is used to check the differences between each root sample group obtained in PCA using the Bray_Curtis distance matrix. **(C)** nitrogen content in roots; **(D)** phosphorus content in roots; **(E)** potassium content in roots; **(F)** calcium content in roots; **(G)** magnesium content in roots; **(H)** iron content in roots **(I)** copper content in roots; **(J)** zinc content in roots; **(K)** manganese content in roots. Values are means ± SE and N = 6 repetitions in each season. According to one-way ANOVA, different letters indicate significant differences between treatments when p<0.05. The significance values of the generalized linear model are as follows: n.s., not significant; *, 0.01<p ≤ 0.05; **, 0.001<p ≤ 0.01; ***, p ≤ 0.001. HR1- healthy plant roots collected in the spring; HR2- healthy plant roots collected in the summer; HR3- healthy plant roots collected in the autumn; HR4- healthy plant roots collected in the winter. DR1- roots of diseased plants collected in the spring; DR2- roots of diseased plants collected in the summer; DR3- roots of diseased plants collected in the autumn; DR4- roots of diseased plants collected in the winter.

The results showed that the lesion area at the base of bamboo stalks of diseased plants in spring was significantly different from that in the other three seasons (**Figure 1A**). At the beginning of the disease, the lesion area at the base of the bamboo stalk in the spring was the smallest. The growth rate of the lesion area in the summer was the fastest, 2.6 times faster than in the spring. In the autumn and winter, the lesion area of diseased plants increased slowly, only by 7.5% and 5%, respectively, and the difference was insignificant. In addition, using principal component analysis (**Figure 1B**), it was found that there were significant differences between the nutrient elements of the roots of healthy and diseased bamboo plants in different seasons.

In the four seasons (**Figures 1C–K**), the contents of N, P, K, Ca, Mg, Fe, Cu, Zn, and Mn in the roots of healthy bamboo plants were higher than those of diseased plants. The season significantly affected the content of N in roots, but there was no significant difference between healthy and diseased plants. In addition, the interaction between different seasons and disease status significantly affected the contents of P, K, Mg, Fe, Cu, Zn, and Mn in the roots, while having no significant effect on the contents of N and Ca.

From spring to summer, the content of P, K, Ca, Mg, Fe, Cu, Zn, and Mn in the roots of both healthy and diseased bamboo plants increased at the fastest rate, with their contents in the roots of healthy bamboo plants in the summer being 2.49 (P), 2.90 (K), 2.71 (Ca), 3.21 (Mg), 4.33 (Fe), 4.44 (Cu), 2.76 (Zn), and 3.08 (Mn) times higher than the content in the roots of healthy bamboo plants in the spring. The nutrient elements in the roots of diseased bamboo plants in the summer were 2.00 (P), 2.49 (K), 2.74 (Ca), 3.83 (Mg), 7.37 (Fe), 3.85 (Cu), 2.78 (Zn), and 2.79 (Mn) times higher than those in the roots of diseased plants in the spring. Meanwhile, there were significant differences in the contents of P, K, Ca, Mg, Fe, Cu, Zn, and Mn between the roots of healthy plants and those of diseased plants in the summer. The contents of nutrient elements in the roots of healthy plants were 1.82 (P), 1.46 (K), 1.23 (Ca), 1.37 (Mg), 1.46 (Fe), 2.40 (Cu), 1.41 (Zn), and 2.10 (Mn) times higher than those in diseased plants in the summer, respectively.

### 3.2 Diversity of root microbial community

**Figure 2:**
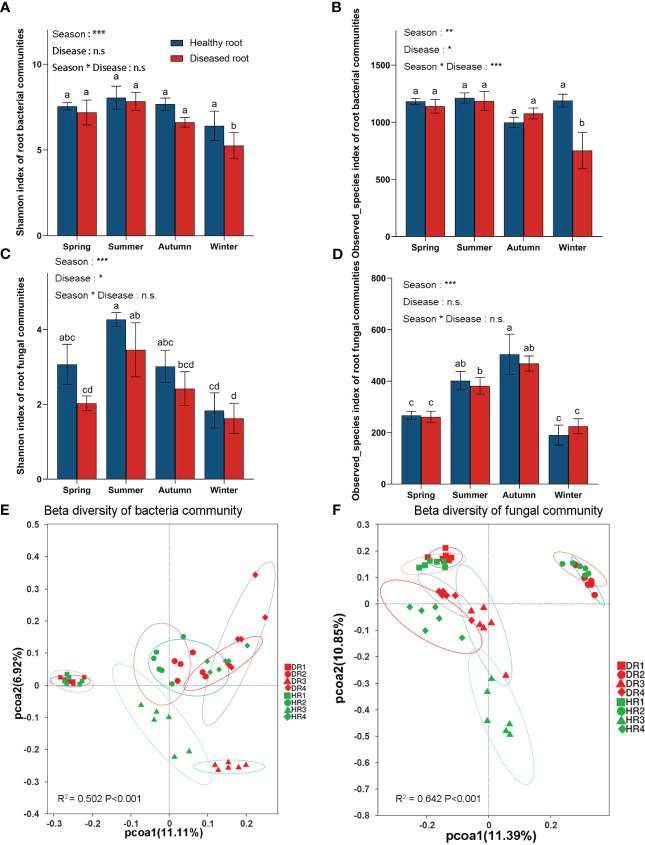
α a diversity index (Shannon and Observed_species index) and β diversity index principal coordinate analysis (PCoA) of bacterial and fungal communities in the roots of healthy and diseased plants sampled in different seasons. **(A)** Shannon index of root bacterial community; **(B)** Observed_species index of root bacterial community; **(C)** Shannon index of root fungal community; **(D)** Observed_species index of root fungi community; **(E)** Principal coordinate analysis (PCoA) diagram of root bacterial community; **(F)** Principal coordinate analysis (PCoA) diagram of root fungal community. One-way similarity analysis (ANOSIM) is used to check the differences between each processed microbial sample group obtained in PCoA using the binary_jaccard distance matrix. Each process was repeated six times. Values are means ± SE and N = 6 repetitions in each season. According to one-way ANOVA, different letters indicate significant differences between treatments when p<0.05. The significance values of the generalized linear model are as follows: n.s., not significant; *, 0.01<p ≤ 0.05; **, 0.001<p ≤ 0.01; ***, p ≤ 0.001. HR1- healthy plant roots collected in the spring; HR2- healthy plant roots collected in the summer; HR3- healthy plant roots collected in the autumn; HR4- healthy plant roots collected in the winter. DR1- roots of diseased plants collected in the spring; DR2- roots of diseased plants collected in the summer; DR3- roots of diseased plants collected in the autumn; DR4- roots of diseased plants collected in the winter.

According to the generalized linear model, the Shannon index, Observed_species index, and Chao1 index (**Figures S3A, B**) showed that seasons significantly affected root bacterial and fungal communities (**Figures 2A–D**). The interaction between season and disease degree affected the diversity of the root bacterial community but did not affect the diversity of the root fungal community. According to one-way ANOVA, the bacterial microbial communities in the roots of healthy plants had no significant differences in different seasons, while the bacterial microbial communities in the roots of diseased plants had significant differences in autumn and winter. In the winter, the Shannon index, Observed_species index, and Chao1 index between healthy plants and diseased plants were significantly different. The fungal microbial communities of healthy and diseased roots varied in different seasons. In the same season, the Shannon index of fungal communities in healthy and diseased plant roots was significant, but the Observed_species index and Chao1 index were not. The Shannon index of root bacterial and fungal communities was highest in the summer.

According to PCoA (**Figures 2E, F**), the microbial communities of healthy bamboo roots and diseased bamboo roots were far apart in different seasons, indicating that different seasons greatly influenced the distribution of microbial communities in roots. However, in the same season (spring, summer, and winter), the bacterial communities of healthy bamboo roots and diseased roots were close to each other and far apart in the autumn, while the fungal communities of healthy bamboo roots and diseased roots were close in the same season. These results showed that the health and disease of plant roots had little effect on microbial community distribution.

### 3.3 Abundance and composition of root microbial groups

In different seasons, the most abundant bacteria in the root bacterial phylum were Proteobacteria (42.56%), Actinobacteria (17.21%), and Acidobacteriota (7.68%), accounting for 67.46% (**Figure S3C**). In the spring and autumn, the relative abundance of Proteobacteria in the roots of healthy plants increased by 2.69% and 5.02%, respectively, compared with that in the roots of diseased plants. In contrast, in the summer and winter, the relative abundance of Actinobacteria in the roots of healthy plants increased by 5.45% and 4.85%, respectively, compared with that in the roots of diseased plants. In the spring, autumn, and winter, the relative abundance of Acidobacteriota in the roots of healthy plants increased by 6.09%, 5.56%, and 4.13%, respectively, but decreased by 0.14% in the summer. The most abundant fungi in the root system in different seasons were Basidiomycota (60.67%) and Ascomycota (28.02%), with both accounting for 88.70% (**Figure S3D**). In the summer, the relative abundance of Basidiomycota in the roots of healthy plants increased by 38.43% compared with the roots of diseased plants. In the spring and winter, the relative abundance of Ascomycota in the roots of healthy plants increased by 18.04% and 25.33%, respectively, compared with the roots of diseased plants.

**Figure 3:**
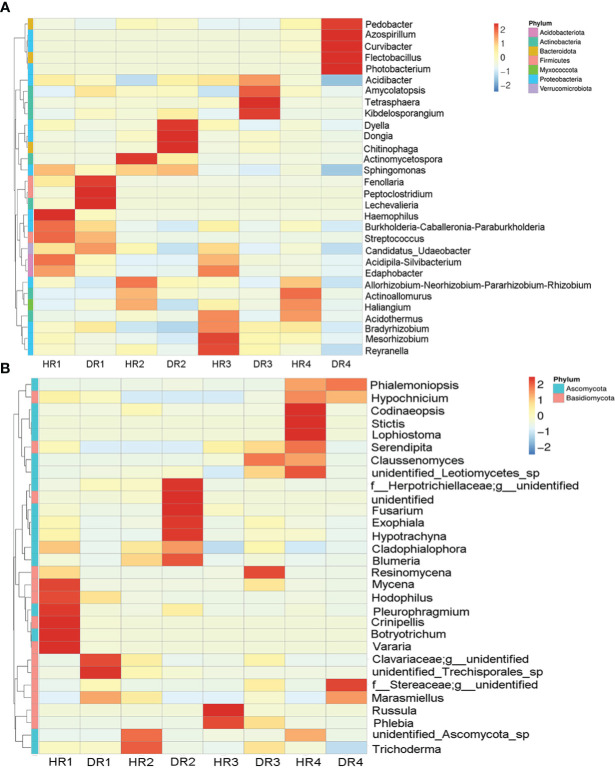
The top thirty genus-level clustering heat maps for bacterial and fungal communities of healthy and diseased plant roots sampled in different seasons. **(A)** Cluster heat map of root bacteria; **(B)** Cluster heat map of root fungi. Heat maps were clustered based on genus level. HR1- healthy plant roots collected in the spring; HR2- healthy plant roots collected in the summer; HR3- healthy plant roots collected in the autumn; HR4- healthy plant roots collected in the winter. DR1- roots of diseased plants collected in the spring; DR2- roots of diseased plants collected in the summer; DR3- roots of diseased plants collected in the autumn; DR4- roots of diseased plants collected in the winter.

According to the heat maps of the top 30 dominant genera, it was found that seasonal changes and pathogen invasion could cause great differences in the abundance of bacteria and fungi in plant roots. The top three dominant bacterial taxa in the roots of healthy bamboo plants were *Bradyrhizobium* sp., *Acidibacter* sp., and *Acidothermus* sp. The top three dominant bacteria genera in the root systems of diseased plants were *Bradyrhizobium* sp., *Dongia* sp., and *Azospirillum* sp. Compared to healthy plants, the relative abundance of *Bradyrhizobium* sp., *Dongia* sp., *Kibdelosporangium* sp., and *Azospirillum* sp. in the roots of diseased plants increased the most in the spring, summer, autumn, and winter, respectively, with increases of 2.40%, 11.00%, 10.60%, and 16.97% (**Figure 3A**). The relative abundance of *Serendipita* sp., *Marasmiellus* sp., and *Blumeria* sp. were the top three dominant genera in the roots of both healthy and diseased plants. Compared with healthy plants, the relative abundance of *Marasmiellus* sp. in the roots of diseased plants increased the most in the spring, autumn, and winter, increasing by 69.68%, 21.26%, and 74.84%, respectively, while decreasing by 43.39% in the summer. Interestingly, during the summer, the relative abundance of *Fusarium* sp. in the roots of diseased plants increased by 7.87% and that of *Trichoderma* sp. decreased by 3.34% (**Figure 3B**).

### 3.4 Significant analysis of differences among root microbial groups, the abundance of *Fusarium*, and the phylogeny of *Fusarium*

**Figure 4:**
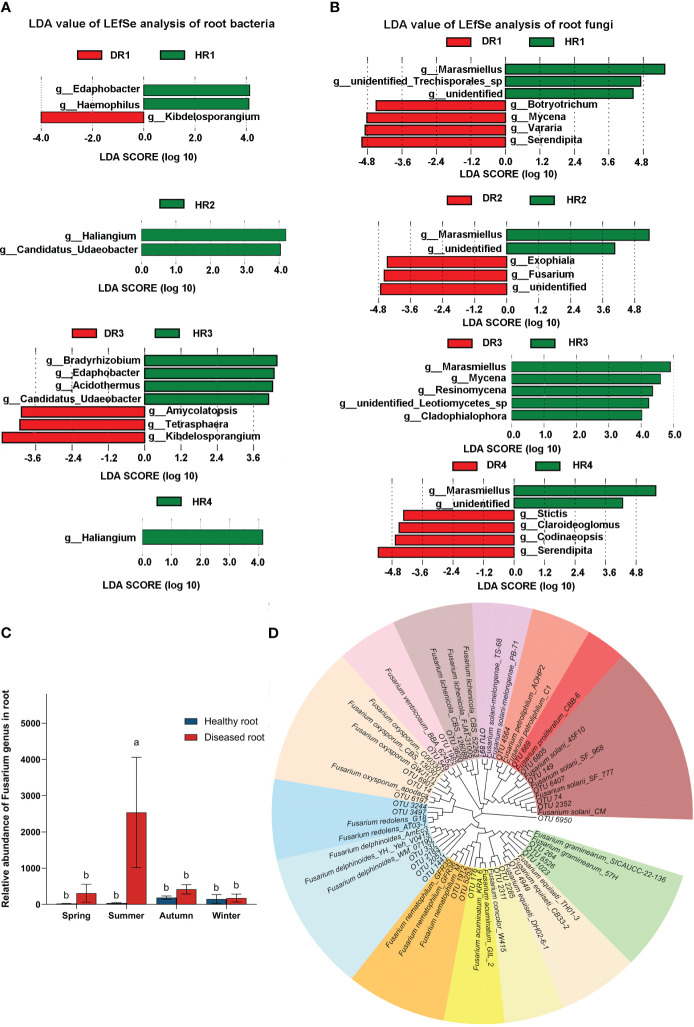
Species analysis of intergroup differences between bacterial and fungal communities of healthy and diseased plant roots sampled in different seasons, the relative abundance of *Fusarium*, and the phylogenetic tree of *Fusarium* in the roots. **(A)** LDA value map of root bacterial community in different seasons; **(B)** LDA value map of root fungal community in different seasons. LDA discriminates the microbial groups that play a significant role in the statistics of multiple groups. The greater the LDA score obtained by LDA analysis (linear regression analysis), the greater the influence of representative species abundance on the different effects. The LDA value chart only shows the taxa that meet the LDA significance threshold > 4.0. **(C)** Relative abundance plots of *Fusarium* in the roots; using one-way ANOVA, different letters indicate significant differences between treatments at p<0.05. **(D)** Phylogenetic tree of *Fusarium* in the roots. Different colors for different species indicate branches of the developmental tree. HR1- healthy plant roots collected in the spring; HR2- healthy plant roots collected in the summer; HR3- healthy plant roots collected in the autumn; HR4- healthy plant roots collected in the winter. DR1- roots of diseased plants collected in the spring; DR2- roots of diseased plants collected in the summer; DR3- roots of diseased plants collected in the autumn; DR4- roots of diseased plants collected in the winter.

There were 36 biomarkers with statistical differences, including 13 genera of root bacteria with significant differences (**Figure 4A**) and 23 genera of root fungi with significant differences (**Figure 4B**). At the genus taxonomic level of bacteria, in the roots of diseased plants, *Kibdelosporangium* sp. was a differential indicator in the spring, and *Amycolatopsis* sp., *Tetrasphaera* sp., and *Kibdelosporangium* sp. were differential indicators in the autumn. At the genus taxonomic level of fungi, in the spring, *Mycena* sp. and *Serendipita* sp. were differential indicators in the roots of diseased plants. In the summer, *Exophiala* sp. and *Fusarium* sp. were differential indicators in the roots of diseased plants. In the winter, *Claroideoglomus* sp. and *Serendipita* sp. were differential indicators in the roots of diseased plants. According to the LDA value, the difference indicator genera of healthy plants and diseased plants in the same season were screened out. By using Deseq2 analysis, the relative abundance ratio of indicator genera between diseased plants and healthy plants showed that nine bacterial indicator genera were positive, four bacterial indicator genera were negative, eight fungal indicator genera were positive, and four fungal indicator genera were negative (**Table S2**, **Table S3**).

In different seasons, the abundance of *Fusarium sp.* in the roots of healthy bamboo plants and diseased bamboo plants was significantly different (P<0.05) (**Figure 4C**). The *Fusarium* abundance in the roots of diseased plants was 13.81, 65.30, 2.22, and 1.22 times higher than that in the roots of healthy bamboo plants in the spring, summer, autumn, and winter, respectively. The *Fusarium* abundance in the roots of diseased plants in the summer was 8.22 times higher than in the spring. The phylogenetic analysis provides abundant information by providing more powerful indications for the possible species or species complexes that the selected OTUs may represent. A total of 30 OTUs at the *Fusarium* level were screened from the root fungal microbial community data, of which only 14 OTUs were sequenced to the species level, while 16 OTUs were only annotated to the *Fusarium* genus. According to the phylogenetic tree (**Figure 4D**), 30 OTUs of the genus *Fusarium* had been annotated to 16 species, among which the pathogen *F. proliferatum* was annotated.

### 3.5 Correlation analysis between the influencing factors and root microbial groups

**Figure 5:**
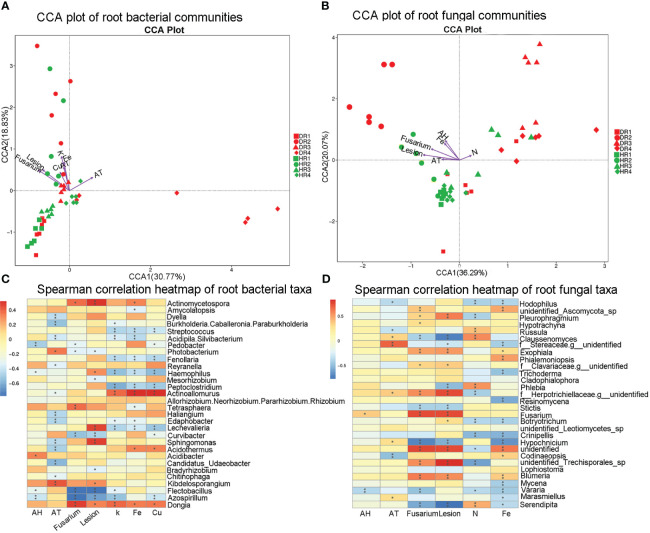
CCA maps and heat maps of the correlation analysis of bacterial and fungal communities with influencing factors in the roots of healthy and diseased plants sampled in different seasons. **(A)** CCA diagram of root bacterial community; **(B)** CCA diagram of root fungal community; **(C)** Spearman analysis heat map of root bacterial community; **(D)** Spearman analysis heat map of root fungal community. Spearman correlation was used to analyze the correlation between influencing factors and the top 30 genera in relative abundance. The Spearman correlation significance values are: *0.01<p ≤ 0.05; ** 0.001<p ≤ 0.01; *** p ≤ 0.001. HR1- healthy plant roots collected in the spring; HR2- healthy plant roots collected in the summer; HR3- healthy plant roots collected in the autumn; HR4- healthy plant roots collected in the winter. DR1- roots of diseased plants collected in the spring; DR2- roots of diseased plants collected in the summer; DR3- roots of diseased plants collected in the autumn; DR4- roots of diseased plants collected in the winter.

The root nutrient elements N, P, K, Ca, Mg, Fe, Cu, and Zn with VIF<20 were screened by VIF analysis. BioENV analysis was used to screen the combination of root nutrient elements with the strongest correlation with the root microbial community. The correlation between nutrient elements K, Fe, and Cu and the root bacterial community was strong (R^2 ^= 0.28), and the correlation between nutrient elements N and Fe and the root fungal community was strong (R^2^ = 0.21). CCA analysis was used to analyze the relationship between the most relevant nutrient element combination, disease incidence index, air temperature, air relative humidity, and root microbial community structure (**Figures 5A, B**). It was found that 49.6% of the root bacterial microbial changes could be explained by the influencing factors, and the nutrient element K (R^2 = ^0.54 P< 0.001) was the dominant influencing factor for the root bacterial microbial community structure. 56.36% of the microbial changes of root fungi could be explained by the influencing factors, in which the lesion area (R^2 = ^0.51 P< 0.001) was the dominant influencing factor for the structure of the root fungal microbiota community.

By Spearman analysis, it was found that *Dongia* sp. and *Actinoallomurus* sp. were significantly positively correlated with K, while *Azospirillum* sp., *Lechevalieria* sp., *Peptoclostridium* sp., *Haemophilus* sp., *Fenollaria* sp., *Acidipila.Silvibacterium* sp., and *Streptococcus* sp. were significantly negatively correlated. At the level of root fungi genus, *Blumeria* sp., *Fusarium* sp., *Trichoderma* sp., *Exophiala* sp., and *Pleurophragmium* sp. were significantly positively correlated with the lesion area, while *Serendipita* sp., *Hypochnicium* sp., *Stictis* sp., *Phlebia* sp., and *Claussenomyces* sp. showed a significant negative correlation (**Figures 5C, D**).

### 3.6 Functional prediction of fungal and bacterial taxa

**Figure 6:**
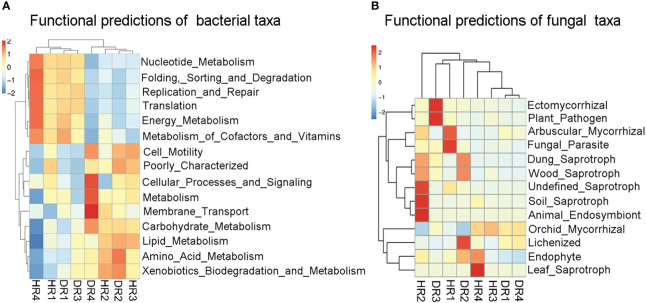
PICRUSt2 and FUNGuild function prediction analysis diagram of bacterial and fungal communities in the roots of healthy and diseased plants sampled in different seasons. **(A)** PICRUSt2 function prediction analysis diagram of root bacterial community. The heat map was clustered based on level 1. **(B)** FUNGuild function prediction analysis diagram of root fungi community. According to a confidence ranking, the prediction of fungal function was annotated at the Highly Probable level. The heat map was clustered based on guild. HR1- healthy plant roots collected in the spring; HR2- healthy plant roots collected in the summer; HR3- healthy plant roots collected in the autumn; HR4- healthy plant roots collected in the winter. DR1- roots of diseased plants collected in the spring; DR2- roots of diseased plants collected in the summer; DR3- roots of diseased plants collected in the autumn; DR4- roots of diseased plants collected in the winter.

As found by PICRUSt2 analysis (**Figure 6A**), Nucleotide_Metabolism, Folding_Sorting_and_Degradation, Replication_and_Repair, Translation, Energy_Metabolism, and Metabolism_of_Cofactors_and_Vitamins were high in relative abundance in the root bacterial community of healthy bamboo plants in the winter, while Cellular_Processes_and_Signaling, Metabolism, Membrane_Transport, and Carbohydrate_Metabolism had a high relative abundance in the roots of diseased plants in the winter, and Amino_Acid_Metabolism had a high relative abundance in the roots of diseased plants in the summer. According to FUNGuild analysis (**Figure 6B**), Arbuscular_Mycorrhizal and Fungal_Parasite had a high relative abundance in the roots of healthy plants in the spring. Undefined_Saprotroph, Soil_Saprotroph, and Animal_Endosymbiont had a high relative abundance in the roots of healthy plants in the autumn. Leaf_Saprotroph had a high relative abundance in the roots of healthy bamboo plants in the winter. Lichenized had a high relative abundance in the roots of diseased plants in the summer. Ectomycorrhizal and Plant_Pathogen had a high relative abundance in the roots of diseased plants in the autumn.

## 4 Discussion

### 4.1 Root pathogens decreased the content of nutrient elements in roots

The nutritional status of plants can act as the primary line of defense against diseases, ultimately determining the susceptibility or resistance of plants to invasive pathogens (Tripathi et al., 2022). In this study, the contents of P, K, Ca, Mg, Fe, Zn, and Mn in the roots of healthy and diseased plants were the highest in the summer and the lowest in the spring. We speculated that the plant grew vigorously in the summer and that the root system absorbed a lot of nutrients for the growth and development of the plant. We observed that nutrient elements such as N, P, K, Ca, Mg, Fe, Cu, Zn, and Mn were lower in the roots of diseased plants sampled in different seasons compared to healthy plants. Previous studies have shown that pathogenic fungi compete with plants for nutrients, which leads to a decrease in nutrient availability and disease resistance due to nutrient deficiency (Graham, 1983). For instance, it has been reported that magnesium deficiency in plants infected with corn stunt spiroplasma CSS and scarcity of zinc lead to pathogenic microorganisms using the host’s metalloprotein as a source of metal (De Oliveira et al., 2002; De Oliveira et al., 2005; Neumann et al., 2017). Furthermore, root necrosis limits plant root growth and nutrient and water absorption (Zhang Y. et al., 2021; Liao et al., 2022). Plant nutrient status plays a significant role in the susceptibility or resistance of plants to invasive pathogens, and certain nutrient elements have been found to affect pathogen invasion and colonization of phloem tissues (Broders et al., 2009). Pathogens can also degrade cell walls or affect membrane permeability, inducing nutritional deficiencies (Fatima and Senthil-Kumar, 2015). A study has found a decrease in the nutrient elements in the fine roots of diseased conifers (Bauch and Schröder, 1982). And the results of this study are consistent with previous studies (Sarkar and Joshi, 1977; Bauch and Schröder, 1982). Pathogens invade the host, which affects the full absorption and utilization of nutrients by plant roots, reduces plant defense abilities, and causes more serious harm from pathogens. In this study, we have not explored how pathogen invasion affects the nutrient content of host roots. We need to further explore the influence of pathogen invasion on plant nutrition in the future.

### 4.2 The pathogen presence correlates with changes in the diversity and structure of the bamboo root microbiome

The study has shown that the diversity of microbial communities in the roots of healthy plants is more abundant than that in diseased plants (Wang et al., 2021). In this study, the Shannon index of healthy and diseased plants was the highest in the summer and the lowest in the winter, indicating that the microbial community diversity of plant roots was the most abundant in the summer. We found that the alpha diversity indices (Shannon index, Observed_species index, and Chao1 index) of bacterial and fungal communities in the roots of healthy plants were higher than those in diseased plants, except for the Observed_species index and Chao1 index of root bacteria in the autumn and fungal communities in the winter.

By comparing the dominant genera in the bacterial and fungal communities between healthy and diseased plant roots collected in different seasons, we found that most of the dominant bacteria in both healthy and diseased roots were beneficial bacteria. For instance, *Bradyrhizobium* sp. and *Azospirillum* sp. are root-stem growth-promoting bacteria that are important in promoting plant growth and development (Ahemad, 2014). *Acidothermus* sp. (Lin et al., 2022; Yang et al., 2022), *Dongia* sp. (Han et al., 2019), and *Kibdelosporangium* sp. (Abd El-Aziz et al., 1997) can improve plant resistance to biotic and abiotic stresses. The beneficial genus *Serendipita* sp. (*S. herbamans*) belongs to the root colonizing fungus, which could compete with the pathogen for niches and inhibit the invasion of the pathogen (Hallasgo et al., 2022). Previous studies have shown that plants invaded by pathogens can use various chemical stimuli to recruit beneficial microorganisms from the environment (Hongwei et al., 2021; Tao et al., 2022). The results of this study are consistent with previous studies. Under the influence of different seasons and pathogen invasion, the community structure of plant root microorganisms was affected, and the beneficial dominant genera in healthy plants and diseased plants were abundant in different seasons.

According to Lefse analysis and Deseq2 analysis, it was found that the relative abundance of differential indicator species in root fungal communities changed more than that of bacterial groups, and the changes of fungal communities and bacterial communities in diseased plants were greater than those in healthy plants. These results indicated that pathogen invasion had a great effect on the community structure of plant root microorganisms, especially on the community structure of fungi (Chen et al., 2020). Through the significant differences between healthy plants and diseased plants, we can screen for beneficial microorganisms that may strengthen the defense ability of plants, increase the disease resistance of plants at the microbial level, and consolidate the micro-ecological balance of plant roots. For example, *Bradyrhizobium* sp. (Siddiqui et al., 2001; Chattopadhyay et al., 2022) and *Amycolatopsis* sp. (Alipour Kafi et al., 2021; Basavarajappa et al., 2023) have the potential to promote plant growth and biocontrol. The beneficial fungus *Serendipita* acts as a differential indicator of diseased plant roots (Mohamed et al., 2020). Ma et al. (2023) found that taxa of differential abundance may play a key role in maintaining plant health, and our findings support previous studies.

The predicted functional survey showed that when the soil-borne pathogen invaded the host, the bacterial community in the root of diseased bamboo mainly carried out amino acid metabolism in the summer. It is speculated that there may be a certain relationship between amino acid metabolism and the occurrence of the disease. Solís-García et al. (2021) found that the enrichment of amino acids may occur in the roots of plants with root rot, and our research results support this study. In healthy plants, the root fungal community was mainly composed of various saprotrophs. Saprophytic fungi can participate in a variety of degradation and metabolic activities. Kusstatscher et al. (2019) found an increase in saprophytic fungi in healthy samples. The function of plant pathogens in the roots of diseased plants was obviously higher than that of healthy plants, which is consistent with the research results of Xie et al. (2022).

### 4.3 The abundance of pathogens in different seasons determined the process of plant diseases

We found that the lesion area in plant roots, the relative abundance of *Fusarium*, and root nutrient elements affected the distribution and structure of the root microbial community. In the spring, the relative abundance of *Fusarium* in the roots of healthy plants and diseased plants was lowest, and in the summer, the relative abundance of *Fusarium* in the roots of diseased plants was highest. In the summer, the percentage of lesion area was positively correlated with the relative abundance of *Fusarium*. A higher abundance of *Fusarium* fungi was associated with a larger necrotic area of plant rhizomes, indicating that the abundance of *Fusarium* fungi was one of the main factors promoting disease development. A phylogenetic tree analysis of *Fusarium* revealed the presence of many *Fusarium* species in hybrid bamboo roots, including *F. proliferatum*, the main pathogen of root rot in hybrid bamboo. Therefore, controlling the abundance of *Fusarium* fungi is crucial to preventing diseases. Previous studies have found that microspore content in soil is significantly positively correlated with the incidence of cotton *Verticillium* wilt. Reducing the quantity of microsclerotia in the soil is the fundamental method to control *Verticillium* wilt (Liu et al., 2018). Liu et al. (2021) found that the abundance of *F. oxysporum* was an important predictor of plant health. Our research supports the results of these previous studies.

### 4.4 The content of K in roots and the lesion area are the key factors influencing the root microbial community

Nutrients and favorable environmental conditions in plants contribute to the high-density growth and reproduction of pathogens, eventually leading to serious diseases. In this study, the lesion area, the relative abundance of *Fusarium*, air temperature, air humidity, and root nutrients affected the distribution and structure of root microbial communities. CCA correlation analysis of the influencing factors and root microbial communities showed that *Fusarium*, lesion area, air humidity, K, Fe, and Cu were all positively correlated with bacterial communities in the roots of healthy and diseased plants in the summer, while *Fusarium*, lesion area, air humidity, air temperature, and Fe were positively correlated with fungal communities in the roots of healthy and diseased plants in the summer. The increase in air temperature and relative humidity in the summer is conducive to the growth of root microorganisms, including the proliferation of pathogens, which results in serious plant disease.

The beneficial *Actinoallomurus* sp. (Inahashi et al., 2015) and *Dongia* sp. could produce active antibacterial substances, which were positively correlated with K. Harmful bacteria, *Peptoclostridium* sp. (Stevenson et al., 2016), *Streptococcus* sp. (Chen et al., 2023), and *Haemophilus* sp. (Myers, 2020), were negatively correlated with K. Potassium plays an important role in plant cell physiology. K is an essential macronutrient that performs critical functions related to enzyme activation, osmoregulation, turgor generation, cell expansion, membrane potential regulation, and pH homeostasis (Ragel et al., 2019). Potassium (K), when present in sufficient concentrations, increases the plant’s polyphenol concentration, which plays a key role in defense mechanisms (Tripathi et al., 2022). The lesion area was significantly positively correlated with the harmful fungi *Blumeria* sp., *Fusarium* sp., and *Exophiala* sp. (Thitla et al., 2022). We speculate that the pathogens invade the host, which leads to damage to the plant’s defense system, and a large number of harmful fungi in the soil invade the host, multiply in the host, destroy the host’s tissue, and lead to the expansion of the lesion area. Our results support the previous studies by Mannaa and Seo (2021) and Popescu et al. (2022).

In summary, the relative abundance of pathogens in plant roots was different in different seasons. The levels of *Fusarium* were the highest in the summer, and the expansion rate of the lesion area was the fastest, resulting in serious disease, while the expansion rate of the lesion area was slow in the autumn and winter. The content of nutrient elements and the composition of the root microbial community were significantly affected by seasonal changes and diseases K and lesion area are dominant influencing factors affecting the composition and structure of root microbial communities. The highest lesion area of rhizomes and the abundance of *Fusarium* in roots were observed during the summer, emphasizing the importance of controlling the abundance of *Fusarium* fungi to prevent diseases.

## Data availability statement

The datasets presented in this study can be found in online repositories. The names of the repository/repositories and accession number(s) can be found below: https://www.ncbi.nlm.nih.gov/genbank/, PRJNA936465 https://www.ncbi.nlm.nih.gov/genbank/, PRJNA936468.

## Author contributions

Conceptualization, LL, YW, and CY; Methodology, LL and YW; Software, LL; Validation, SYL, TZ, and SH; Formal Analysis, LL and YW; Investigation, TL and CY; Resources, TL; Data Curation, CY; Writing–Original Draft Preparation, LL; Writing–Review and Editing, SJL and TL; Visualization, LL; Supervision, SH; Project Administration, TZ; Funding Acquisition, SJL. All authors contributed to the article and approved the submitted version.

## Supplementary material

The raw data of 16S rRNA totaled 4,483,656, and 3,093,359 valid sequences were obtained after quality control, splicing, and chimera removal; the raw data of ITS totaled 4,504,231 and 3,451,386 valid sequences. The rarefaction curves of all samples tended to be flat, and the number of OTU features of samples would not increase sharply with the increase in sequencing number, indicating that the sequencing depth was sufficient to reflect the species diversity of samples (**Figure S2**). Bacterial (n = 8401) and fungal (n = 3897) OTUs were isolated from bamboo root microorganisms in different seasons. There were 632 species in 66 phyla, 98 classes, 222 orders, 407 families, and 1080 genera in bacterial community OTUs, and 1373 species in 16 phyla, 66 classes, 180 orders, 424 families, and 848 genera in fungal community OTUs. According to the Venn diagram of each sample obtained from OTUs (**Figures S2C–H**), there were 660 bacterial OTUs in common in 48 samples, 971 OTUs in healthy plant root samples, and 800 OTUs in diseased plant root samples. There were 84 fungal OTUs in common in 48 samples, 143 OTUs in healthy plant root samples, and 149 OTUs in diseased plant root samples.

Supplementary Figure 1 Sample plot setting and pictures of healthy and diseased plants collected in different seasons. **(A)** Sampling plots of healthy plants and diseased plants; **(B)** Healthy plants collected in spring; **(C)** Healthy plants collected in summer; **(D)** Healthy plants collected in autumn; **(E)** Healthy plants collected in winter; **(F)** Diseased plants collected in spring; **(G)** Diseased plants collected in summer; **(H)** Diseased plants collected in autumn; **(I)** Diseased plants collected in winter.

Supplementary Figure 2 Rarefaction curves, Petal, and Venn diagrams of bacterial and fungal communities in the roots of healthy plants and diseased plants sampled in different seasons. **(A)** Rarefaction curve of root bacteria; **(B)** Rarefaction curve of root fungi. **(C)** Petal diagram of root bacterial communities; **(D)** Venn diagram of root bacterial communities of healthy plants; **(E)** Venn diagram of root bacterial communities of diseased plants; **(F)** Petal diagram of root fungal communities; **(G)** Venn diagram of root fungal communities of healthy plants; **(H)** Venn diagram of root fungal communities of diseased plants. HR1- healthy plant roots collected in spring; HR2- healthy plant roots collected in summer; HR3- healthy plant roots collected in autumn; HR4- healthy plant roots collected in winter. DR1- roots of diseased plants collected in spring; DR2- roots of diseased plants collected in summer; DR3- roots of diseased plants collected in autumn; DR4- roots of diseased plants collected in winter.

Supplementary Figure 3 α diversity index (Chao 1 index) and top 10 phylum-level relative abundance maps for bacterial and fungal communities of healthy and diseased plant roots sampled in different seasons. **(A)** Chao 1 index of root bacterial community; **(B)** Chao 1 index of root fungal community; **(C)** Phylum-level relative abundance maps for root bacteria; **(D)** Phylum-level relative abundance maps for root fungi. Each process was repeated six times. Values are means ± SE and N = 6 repetitions in each season. According to one-way ANOVA, different letters indicate significant differences between treatments when p<0.05. The significance values of the generalized linear model are as follows: n.s., not significant; *, 0.01<p ≤ 0.05; **, 0.001<p ≤ 0.01; ***, p ≤ 0.001. Fungal and bacterial communities not identified to phylum taxa were assigned to “other “. HR1- healthy plant roots collected in spring; HR2- healthy plant roots collected in summer; HR3- healthy plant roots collected in autumn; HR4- healthy plant roots collected in winter. DR1- roots of diseased plants collected in spring; DR2- roots of diseased plants collected in summer; DR3- roots of diseased plants collected in autumn; DR4- roots of diseased plants collected in winter.
