# Supplementary material for: Seasonal changes in the abundance Fusarium proliferatium, microbial endophytes and nutrient levels in the roots of hybrid bamboo Bambusa pervariabilis × Dendrocalamopsis grandis
Source: Front Plant Sci. 2023 Jul 19;14:1185449. doi: 10.3389/fpls.2023.1185449 (PMC10394707; doi:10.3389/fpls.2023.1185449)
Supplement: Supplementary file 1 [file DataSheet_1.pdf]

Table S1 The air temperature and humidity of the sampling site in different seasons

| Season | Air temperature(℃) | Air humidity(%) |
|---|---|---|
| Spring | 23.3 | 77 |
| Summer | 31.4 | 80 |
| Autumn | 25.1 | 73 |
| Winter | 8.2 | 70 |

Table S2 Differential abundance of indicator bacteria genera between diseased plants and healthy plants in the same season (DeSEQ2 test).

| Group | Taxa | Log2 Fold Change DR vs. HR (SE) | Test statistic | p-value |
|---|---|---|---|---|
| Spring | *Edaphobacter*_sp | 1.76 (0.43) | 4.11 | <0.01* |
| | *Haemophilus*_sp | 8.43 (2.34) | 3.61 | <0.01* |
| | *Kibdelosporangium*_sp | -2.79 (0.68) | -4.14 | <0.01* |
| Summer | *Haliangium*_sp | 3.87 (1.66) | 2.33 | 0.02* |
| | Candidatus_*Udaeobacter*_sp | 5.14 (1.53) | 3.37 | <0.01* |
| | *Bradyrhizobium*_sp | 0.71 (0.50) | 1.41 | 0.16 |
| Autumn | *Edaphobacter*_sp | 2.99 (0.65) | 4.63 | <0.01* |
| | *Acidothermus*_sp | 4.33 (0.85) | 5.08 | <0.01* |
| | *Candidatus_Udaeobacter*_sp | 6.45 (1.12) | 5.78 | <0.01* |
| | *Amycolatopsis*_sp | -4.24 (0.51) | -8.33 | <0.01* |
| | *Tetrasphaera*_sp | -3.51 (0.87) | -4.05 | <0.01* |
| | *Kibdelosporangium*_sp | -1.28 (3.12) | -0.41 | 0.68 |
| Winter | *Haliangium*_sp | 2.65 (1.50) | 1.77 | 0.08 |

*$p$<0.05, significant.

Table S3 Differential abundance of indicator fungus genera between between diseased plants and healthy plants in the same season (DeSEQ2 test).

| Group | Taxa | Log2 Fold Change DR vs. HR(SE) | Test statistic | *p*-value |
|---|---|---|---|---|
| Spring | *Marasmiellus*_sp | -4.5 (1.33) | -3.39 | <0.01* |
| | *Mycena*_sp | 6.83 (1.49) | 4.59 | <0.01* |
| | *Serendipita*_sp | 6.86 (1.29) | 5.32 | <0.01* |
| Summer | *Marasmiellus*_sp | 13.02 (0.94) | 13.79 | <0.01* |
| | *Exophiala*_sp | 3.62 (1.71) | 2.11 | 0.03* |
| | *Fusarium*_sp | 4.26 (3.02) | 1.41 | <0.01* |
| Autumn | *Marasmiellus*_sp | -2.64 (1.66) | -1.59 | 0.11 |
| | *Mycena*_sp | 0.18 (3.10) | 0.03 | 0.95 |
| | *Cladophialophora*_sp | 0.55 (3.10) | 0.18 | 0.86 |
| Winter | *Marasmiellus*_sp | -1.3 (3.10) | -0.42 | 0.68 |
| | *Claroideoglomus*_sp | -0.97 (3.09) | -0.31 | 0.75 |
| | *Serendipita*_sp | 3.34 (1.47) | 2.26 | 0.02* |

*$p<0.05$, significant.
