# Supplementary figures and images for: Seasonal changes in the abundance Fusarium proliferatium, microbial endophytes and nutrient levels in the roots of hybrid bamboo Bambusa pervariabilis × Dendrocalamopsis grandis
Source: Front Plant Sci. 2023 Jul 19;14:1185449. doi: 10.3389/fpls.2023.1185449 (PMC10394707; doi:10.3389/fpls.2023.1185449)

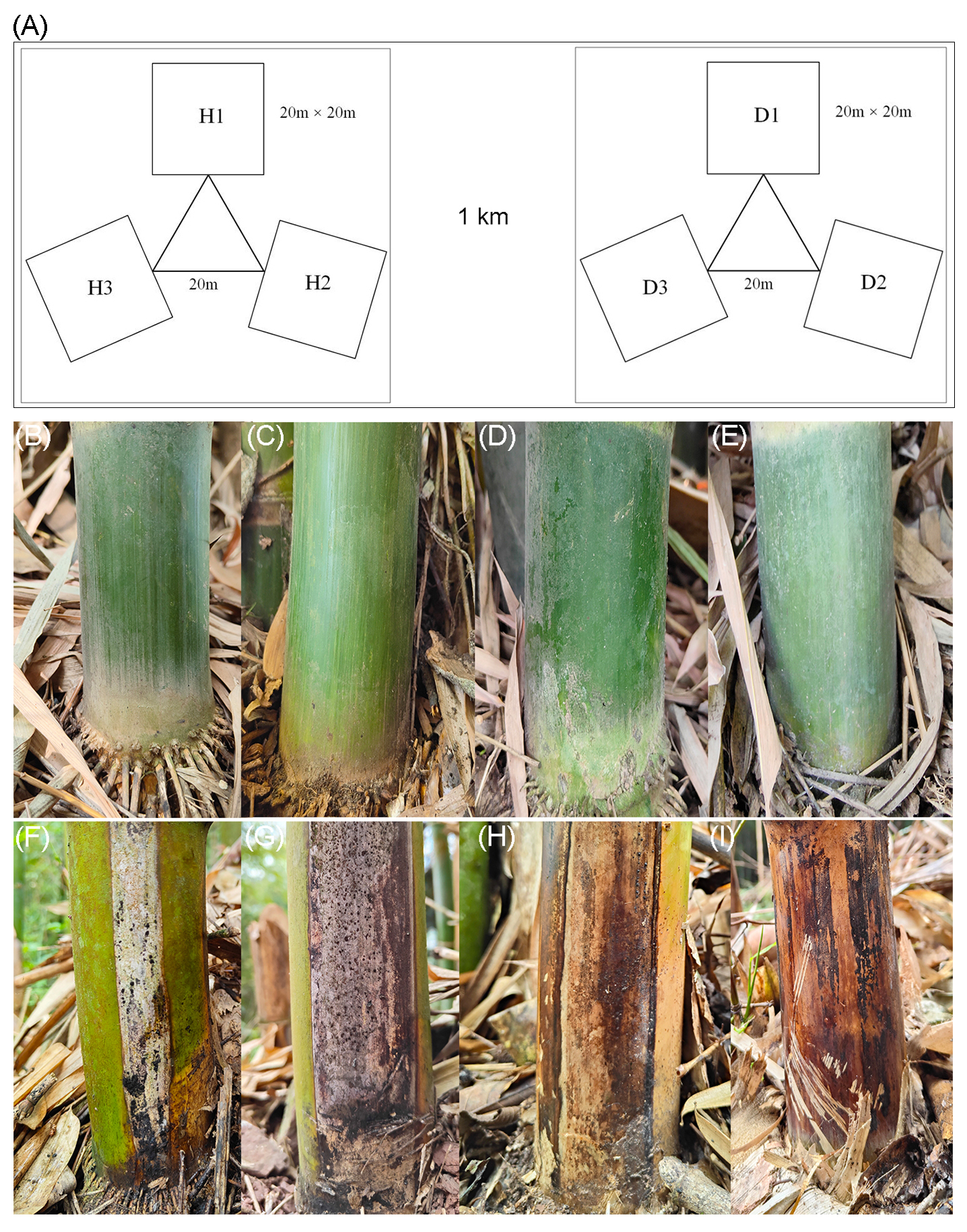

Supplement: Supplementary Figure 1 — Sample plot setting and pictures of healthy and diseased plants collected in different seasons. (A) Sampling plots of healthy plants and diseased plants; (B) Healthy plants collected in spring; (C) Healthy plants collected in summer; (D) Healthy plants collected in autumn; (E) Healthy plants collected in winter; (F) Diseased plants collected in spring; (G) Diseased plants collected in summer; (H) Diseased plants collected in autumn; (I) Diseased plants collected in winter. [file Image_1.jpeg]

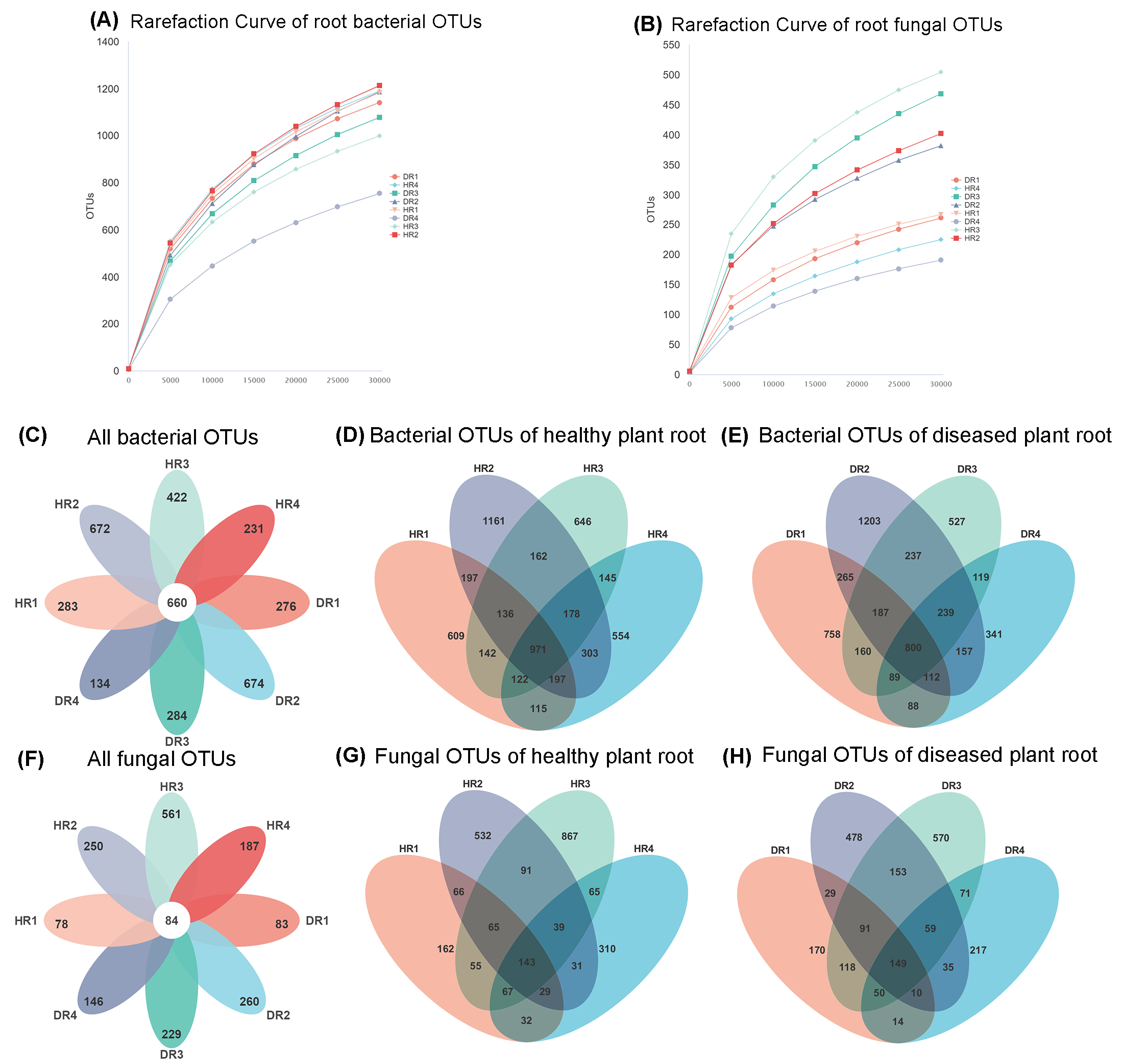

Supplement: Supplementary Figure 2 — Rarefaction curves, Petal, and Venn diagrams of bacterial and fungal communities in the roots of healthy plants and diseased plants sampled in different seasons. (A) Rarefaction curve of root bacteria; (B) Rarefaction curve of root fungi. (C) Petal diagram of root bacterial communities; (D) Venn diagram of root bacterial communities of healthy plants; (E) Venn diagram of root bacterial communities of diseased plants; (F) Petal diagram of root fungal communities; (G) Venn diagram of root fungal communities of healthy plants; (H) Venn diagram of root fungal communities of diseased plants. HR1- healthy plant roots collected in spring; HR2- healthy plant roots collected in summer; HR3- healthy plant roots collected in autumn; HR4- healthy plant roots collected in winter. DR1- roots of diseased plants collected in spring; DR2- roots of diseased plants collected in summer; DR3- roots of diseased plants collected in autumn; DR4- roots of diseased plants collected in winter. [file Image_2.jpeg]

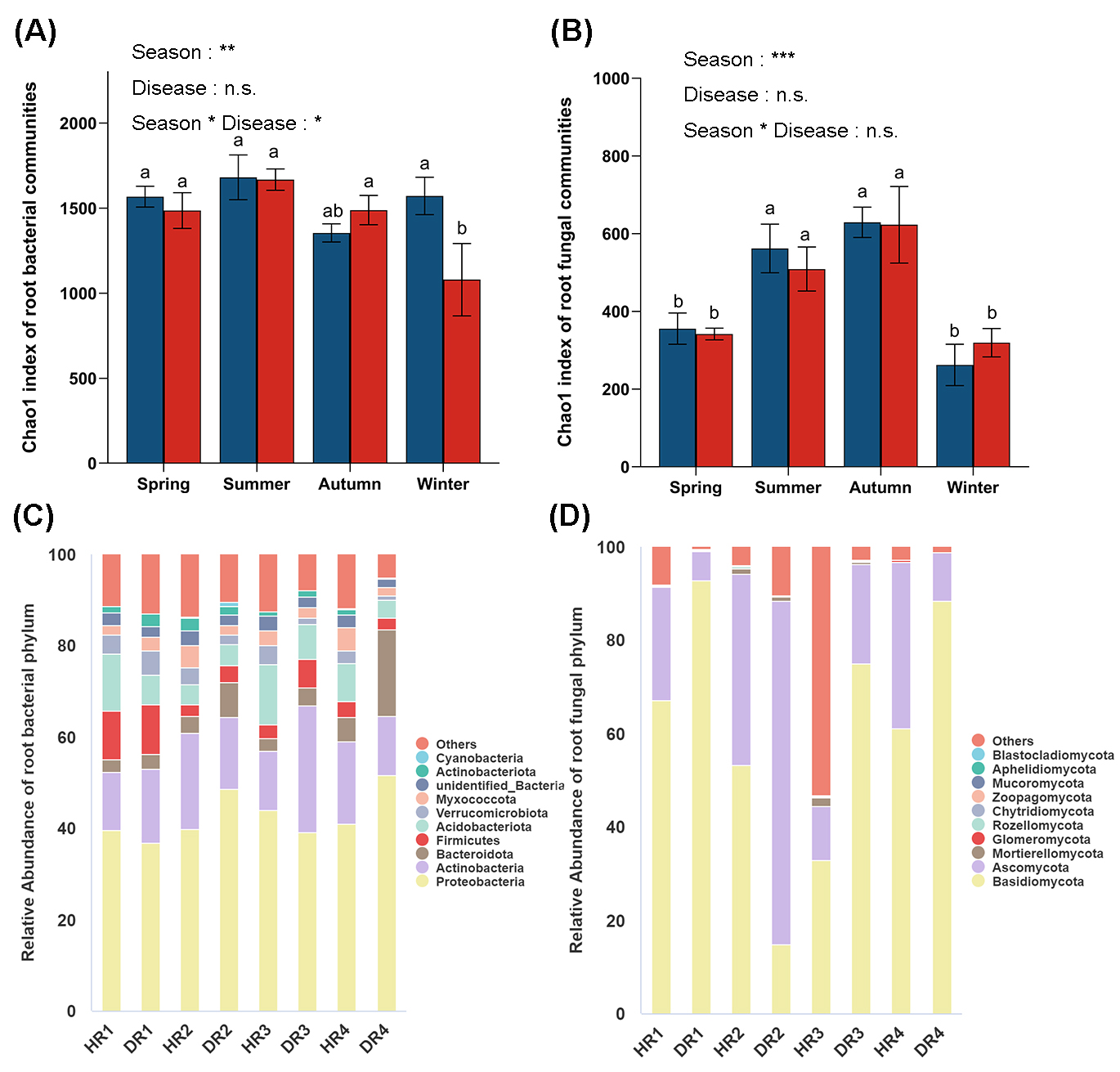

Supplement: Supplementary Figure 3 — α diversity index (Chao 1 index) and top 10 phylum-level relative abundance maps for bacterial and fungal communities of healthy and diseased plant roots sampled in different seasons. (A) Chao 1 index of root bacterial community; (B) Chao 1 index of root fungal community; (C) Phylum-level relative abundance maps for root bacteria; (D) Phylum-level relative abundance maps for root fungi. Each process was repeated six times. Values are means ± SE and N = 6 repetitions in each season. According to one-way ANOVA, different letters indicate significant differences between treatments when p<0.05. The significance values of the generalized linear model are as follows: n.s., not significant; *, 0.01<p ≤ 0.05; **, 0.001<p ≤ 0.01; ***, p ≤ 0.001. Fungal and bacterial communities not identified to phylum taxa were assigned to “other “. HR1- healthy plant roots collected in spring; HR2- healthy plant roots collected in summer; HR3- healthy plant roots collected in autumn; HR4- healthy plant roots collected in winter. DR1- roots of diseased plants collected in spring; DR2- roots of diseased plants collected in summer; DR3- roots of diseased plants collected in autumn; DR4- roots of diseased plants collected in winter. [file Image_3.jpeg]
